# Prospective Questionnaire Survey on Adherence to Oral 5‐Aminosalicylic Acid in Patients With Ulcerative Colitis

**DOI:** 10.1002/jgh3.70259

**Published:** 2025-08-21

**Authors:** Tsuyoshi Beppu, Fumihito Hirai, Kensei Ohtsu, Masahiro Kishi, Teruyuki Takeda, Shigeyoshi Yasukawa, Noritaka Takatsu, Takashi Hisabe, Toshiharu Ueki, Kenshi Yao

**Affiliations:** ^1^ Inflammatory Bowel Disease Center, Fukuoka University Chikushi Hospital Chikushino Fukuoka Japan; ^2^ Department of Gastroenterology Matsusaka Chuo General Hospital Matsusaka Mie Japan; ^3^ Department of Gastroenterology Fukuoka University Faculty of Medicine Fukuoka Fukuoka Japan; ^4^ Department of Gastroenterology Fukuoka University Chikushi Hospital Chikushino Fukuoka Japan; ^5^ Department of Endoscopy Fukuoka University Chikushi Hospital Chikushino Fukuoka Japan

**Keywords:** 5‐aminosalicylic acid, adherence, ulcerative colitis

## Abstract

**Background:**

Adherence to 5‐aminosalicylic acid (5‐ASA), an anchor drug for ulcerative colitis (UC), affects remission maintenance. This study aimed to investigate the current status of adherence to 5‐ASA and identify the factors associated with adherence.

**Methods:**

We enrolled UC patients whose primary maintenance therapy was oral 5‐ASA at our hospital between January 2017 and December 2018. The adherence status was examined using a questionnaire survey. Various factors, including patient background and disease activity, were also analyzed to identify those related to adherence. A second questionnaire with the same content was performed 1 year after the initial survey for comparison.

**Results:**

In total, 297 patients with UC answered the first questionnaire, of which 278 answered the second questionnaire. Complete adherence rates at the time of the first and second questionnaires were 49.8% (148/297) and 55.8% (155/278), respectively. Full‐time employment (*p* = 0.00893), eating one or two meals per day (*p* = 0.00641), and multiple daily doses (*p* = 0.0153) were significantly associated with lower adherence. The 119 patients who fully adhered to both the primary and secondary questionnaires had a significantly higher cumulative non‐relapse rate at 1 year than those 159 who were not (85.7% vs. 76.1%, *p* = 0.0207).

**Conclusions:**

UC patients with high adherence to 5‐ASA showed a high rate of remission maintenance, so adherence should be improved by considering factors such as the number of medications taken, work status, and dietary habits.

## 1 Introduction

Ulcerative colitis (UC) is a nonspecific inflammatory bowel disease of unknown etiology that causes erosions and ulcers in the colon and usually shows a relapsing course. Preparations of 5‐aminosalicylic acid (5‐ASA) play a central role in the treatment of patients with mildly to moderately active UC and those in remission [1, 2]. Adherence to 5‐ASA preparations is a major factor that affects therapeutic outcomes, and many reports on this topic have been published [3, 4, 5, 6, 7, 8, 9, 10, 11, 12, 13, 14, 15, 16, 17, 18, 19, 20]. However, the methodology of analysis differs between these reports, and the adherence rate results are inconsistent [3, 4, 5]. However, almost all studies have pointed out that non‐adherence to 5‐ASA is the main risk factor for relapse in remission maintenance therapy [3, 6]. It has also been reported that the COVID‐19 pandemic has resulted in a significant number of IBD patients with poor medication adherence and many patients with stable symptoms postponing medical visits [21]. In addition, relapse due to non‐adherence is detrimental to healthcare costs because it prompts unscheduled medical visits, including emergency unit care, additional treatment, and hospitalization [7, 8].

Therefore, improving adherence in patients with UC receiving 5‐ASA maintenance remission therapy is of critical clinical importance. Clarifying the factors contributing to non‐adherence is key for improving 5‐ASA adherence and contributing to healthcare cost reduction. Risk factors for non‐adherence have been reported to include factors directly related to treatment, such as the number of medications taken and understanding the need for treatment, as well as factors affecting patient characteristics, such as sex, educational background, and employment or education status [9, 10, 11, 12, 13, 14, 15, 16, 17, 18, 19, 20]. In particular, it has been noted that multiple doses of 5‐ASA preparations are clearly more likely to result in non‐adherence than single doses. However, most studies have only evaluated adherence at a single time point; few have evaluated adherence across a number of doses over time. In addition, patients in previous reports received other drugs, such as steroid immunomodulators and biological agents, and it is unclear whether adherence to 5‐ASA purely affects disease activity [7, 17]. Therefore, in this study, we conducted a two‐part questionnaire survey on adherence among outpatients with UC for whom 5‐ASA is the mainstay of remission maintenance treatment to determine the current status of 5‐ASA agent adherence and identify risk factors for non‐adherence. This study aimed to determine the current status of adherence to 5‐ASA and identify risk factors for non‐adherence by investigating medication regimens and patient backgrounds. Moreover, we verified whether persistently high adherence had a positive effect on therapeutic efficacy over time.

## 2 Patients and Methods

### 2.1 Study Design and Subjects

This study was a 1‐year prospective cohort study of outpatients with UC. A total of 824 UC patients were diagnosed with UC and attended the outpatient clinic of the Fukuoka University Chikushi Hospital between January 2017 and December 2018. Of these, 289 patients who were receiving biologics, calcineurin inhibitors, prednisolone (20 mg/day), study drugs, and cytapheresis were excluded to focus on the remission‐maintaining effects of 5‐ASA. The remaining 535 patients were eligible for this study.

### 2.2 Questionnaire Survey

The primary questionnaire survey began in January 2017 and ended in December 2018. The secondary questionnaire survey was administered 1 year later to patients who had answered the primary questionnaire. This investigation was explained to the patient by the attending physician. The completed questionnaires were collected on the day of the consent for the survey or on the day of the next visit. Due to differences in the intervals between outpatient visits, the second survey was not exactly 1 year later, but it was within 1 year ± 1 month. There was no specific intervention by medical staff between the primary and secondary surveys. Patients received usual care from their gastroenterologist, and treatment varied according to their clinical judgment. Patient adherence rates were not reported to physicians.

The content of the questionnaire included information on schooling and adherence, dietary, and employment status, medications, and methods of administration and was a self‐administered questionnaire survey without intervention by medical staff (Table S1).

### 2.3 Patients Background, Laboratory Data, and Activity Indices

Patient background (sex, age, disease duration), clinical symptoms, and blood tests (white blood cells, hemoglobin, platelets, albumin, C‐reactive protein, and erythrocyte sedimentation rate) were obtained from electronic medical records at the time of the survey. To evaluate disease activity, the number of bowel movements, degree of bloody stools, and partial disease activity index score (p‐DAI), which excluded mucosal appearance sub scores of the DAI (also known as the Sutherland index) [22], were investigated. P‐DAI was calculated based on the stool frequency sub score, rectal bleeding sub score, and physician's rating of disease activity [23].

### 2.4 Adherence

Adherence to oral 5‐ASA was defined as taking the drug daily without failure, and non‐adherence was defined as the rest. Cases that were adherent in both the primary and secondary questionnaires were defined as the continuous adherence group, and all other cases were defined as the non‐continuous adherence group. Clinical relapse was defined as the need for additional induction therapy (including dose escalation of oral 5ASA), excluding an additional dose of topical therapy, during follow‐up 1 year after the initial survey. Surveys were not conducted at the time of relapse; adherence at the time of relapse was unknown.

### 2.5 Endpoints

The primary endpoint was the rate of adherence to each primary and secondary questionnaire. Secondary endpoints included: (1) factors affecting 5‐ASA adherence, (2) changes in adherence and factors affecting adherence based on the results of both questionnaires, and (3) a comparison of the cumulative non‐relapse rate between the continuous adherent group and the non‐continuous adherent group.

This study was approved by the Clinical Research Review Committee of the Fukuoka University. The questionnaire and other information were explained to the patients at an outpatient clinic, and written informed consent was obtained. The study was registered in the University Hospital Medical Information Network Clinical Trial Registry (UMIN‐CTR) (UMIN000035499).

### 2.6 Statistical Analysis

Continuous variables are expressed as mean ± standard deviation for each measurement. Frequency comparisons were made using the chi‐square test or Fisher's exact test, and t‐tests were used to compare variables between the two groups. Logistic regression analysis was used to obtain multivariate‐adjusted odds ratios and 95% confidence intervals (CIs) for patient background factors affecting adherence. The cumulative non‐relapse rates for the continuous and non‐continuous adherent groups were calculated using the Kaplan–Meier method and compared between groups using the log‐rank test. Statistical significance was set at *p* < 0.05. All statistical analyses were performed using EZR version 1.33 (Saitama Medical Center, Jichi Medical University, Saitama, Japan) [24].

## 3 Results

### 3.1 Response Rate to Questionnaires and Patient Backgrounds

**FIGURE 1:**
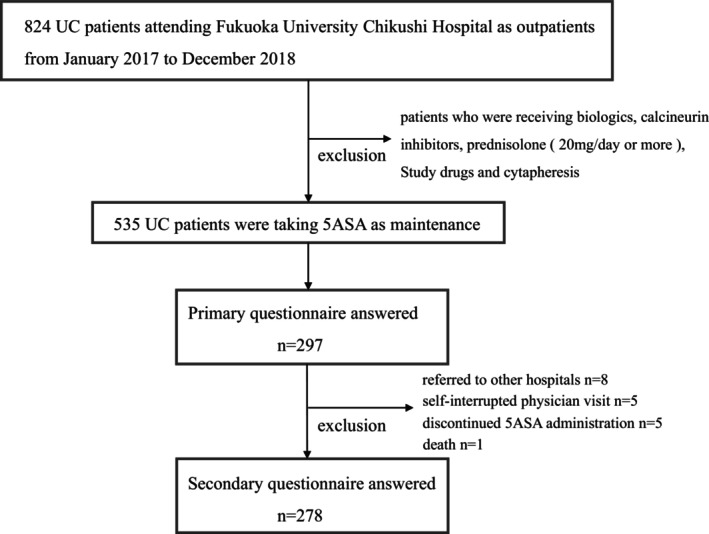
Flowchart of subjects. Of the 535 patients, the primary questionnaire was answered on 297 patients (55.5%), and the secondary questionnaire was answered on 278 patients 1 year later.

The primary questionnaire was answered by 297 (55.5%) of the 535 eligible patients. Of these subjects, 125 patients (42.1%) were female and 172 patients (57.9%) were male, with a mean age of 45.8 ± 16.0 years. The site of involvement was total colitis in 149 patients (50.2%), left‐sided colitis in 119 patients (40.1%), and proctitis in 29 patients (9.8%). The partial DAI was 0.73 ± 1.15 (Table 1). The secondary questionnaire was answered by 278 (93.6%) of the 297 patients who completed the primary questionnaire, excluding 8 patients who were referred to other hospitals, 5 patients who self‐interrupted medical visits, 5 patients who discontinued 5 ASA administration, and 1 patient who died of a disease other than IBD (Figure 1).

**TABLE 1:** Characteristics of ulcerative colitis patients (primary questionnaire) (*n* = 297).

Sex (male: female)	172:125
Age (mean ± SD, years)	45.8 ± 16.0
Disease duration (> 5 years: ≤ 5 years)	84:213
Disease type (pancolitis/left—sided colitis/proctitis)	149:119:29
Partial Disease Activity Index score (mean ± SD, years)	0.73 ± 1.15
Employment status (student: full‐time employment: hourly work: other)	16:162:37:82
Meal status (always eat 3 meals: sometimes skip meals)	217:80
Number of drugs taken (1 type:2 types:3 types:4 types or more)	98:81:44:74
Types of 5‐ASA formulations	
Asacol: Pentasa: Salazopyrin: Lialda	148:131:12:6
Dosage form of 5‐ASA formulation (tablet: granule)	232:65
Number of single doses of 5‐ASA formulation	
1 tablet/packet: 2 tablets/packets: 3 tablets/packets: 4 tablets/packets	64:98:88:47
5ASA number of doses per day (1 time:2 times:3 times or more)	60:109:128

*Note:* Values are presented as mean ± SD or number.

Abbreviations: 5‐ASA, 5‐aminosalicylic acid; SD, standard deviation.

### 3.2 Adherence Rates at the Time of the Primary and Secondary Questionnaires

**FIGURE 2:**
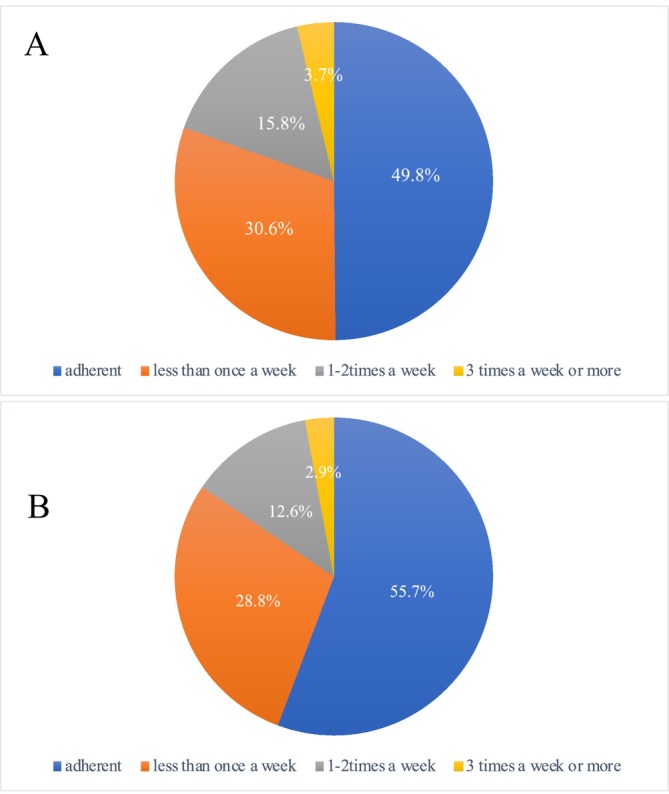
Adherence rates at the time of the primary questionnaire. The adherence rate was 49.8% (148/297) (A), Adherence rates at the time of the secondary questionnaire. The adherence rate was 55.8% (155/278) (B).

Figure 2 shows the adherence rates for the primary and secondary questionnaires. Adherence rates for the primary and secondary questionnaires were 49.8% (148/297) and 55.7% (155/278), respectively. Adherence rates for the secondary questionnaire were slightly higher; although the comparison between the two did not show a significant difference.

### 3.3 Factors Influencing Adherence to 5‐ASA

Table 2 shows a comparison of the patient backgrounds and details of 5‐ASA medication in the primary questionnaire between the adherent (*n* = 148) and non‐adherent groups (*n* = 149). On the univariate analysis, full‐time employment (*p* = 0.00737), eating one or two meals per day (*p* = 0.00252), tablet formulation (*p* = 0.0173), and multiple daily doses of 5‐ASA (*p* = 0.0005) were more common in the non‐adherent group. Table S2 shows a comparison of disease activity and blood test data; there were no significant differences between the two groups. Similar comparisons were made for the secondary questionnaire with similar results (Tables S3 and S4).

**TABLE 2:** Comparison of patient backgrounds and items related to 5‐ASA preparations in the Adherent group and Non‐adherent group at the time of the primary questionnaire.

Factors	Adherent group (*n* = 148)	Non‐adherent group (*n* = 149)	*p*
Gender (Male: Female)	88:60	84:65	0.639
Age			
Under 40:Over 40	48:100	64:85	0.0726
Disease duration			
Less than 5 years:5 years or more	41:107	43:106	0.898
Disease type			
Pancolitis type: Others	80:68	69:80	0.202
Working situation			
Full‐time employment: Others	69:79	93:56	0.00737
Eating habits			
3 meals a day:1–2 meals a day	120:28	97:52	0.00252
Number of medications taken			
Less than 4 types: 4 types or more	105:43	118:31	0.109
5‐ASA formulation			
Dosage form (Tablet: Granule)	107:41	125:24	0.0173
Number of tablets per dose			
Less than 3 tablets/packets: 3 tablets/packets or more	77:71	85:64	0.416
Number of doses per day			
Single dose: Multiple doses	42:106	18:131	0.0005

*Note:* Values are presented as number.

Abbreviation: 5‐ASA, 5‐aminosalicylic acid.

Based on the results of the primary questionnaire, a multivariate logistic regression analysis was conducted by adding items with significant differences, the number of medications with significant trends, age, and sex. The results showed that factors affecting adherence included multiple daily dosing of 5‐ASA [odds ratio (OR):2.39; 95% CI: 1.18–4.82, *p* = 0.0153], eating one or two meals a day (OR:2.19; 95% CI: 1.25–3.85, *p* = 0.00641), and full‐time employment (OR:2.01; 95% CI: 1.19–3.41, *p* = 0.00893) were identified as independent factors (Table 3). A similar multivariate analysis using logistic regression was conducted on the secondary questionnaire, and similar results were obtained (Table S5).

**TABLE 3:** Multivariate analysis of factors related to adherence during the primary questionnaire.

Factors	Odds ratio	95% CI	*p*
Number of daily doses of 5‐ASA preparations multiple doses	2.39	1.18–4.82	0.0153
Eating habits—1–2 meals a day	2.19	1.25–3.85	0.00641
Working situation—full time employment	2.01	1.19–3.41	0.00893
Number of medications taken—4 or more	0.76	0.42–1.37	0.358
Age—40+	0.72	0.42–1.22	0.223
Gender—female	1.52	0.90–2.58	0.116
5‐ASA dosage form granules	0.77	0.39–1.52	0.454

Abbreviations: 5‐ASA, 5‐aminosalicylic acid; CI, confidence interval.

### 3.4 Changes in Adherence Over Time and Its Factors

As mentioned above, adherence rates in the secondary questionnaire were higher than those in the primary questionnaire; therefore, we compared patient backgrounds, details of taking 5‐ASA, disease activity, and laboratory data at the time of completing the two questionnaires. The proportion of patients receiving a single dose of 5‐ASA was significantly higher in the secondary questionnaire (29.5%) than in the primary questionnaire (20.2%) (*p* = 0.0118). In particular, the adherence rates significantly improved from 41.7% to 72.2% in the 36 patients who switched from multiple doses to a single dose of 5‐ASA (*p* = 0.01679) (Figure S1). No significant differences were observed in any other parameters (data not shown).

### 3.5 Comparison of Cumulative Non‐Recurrence Rates by Differences in Mid‐Term Adherence to 5‐ASA

**FIGURE 3:**
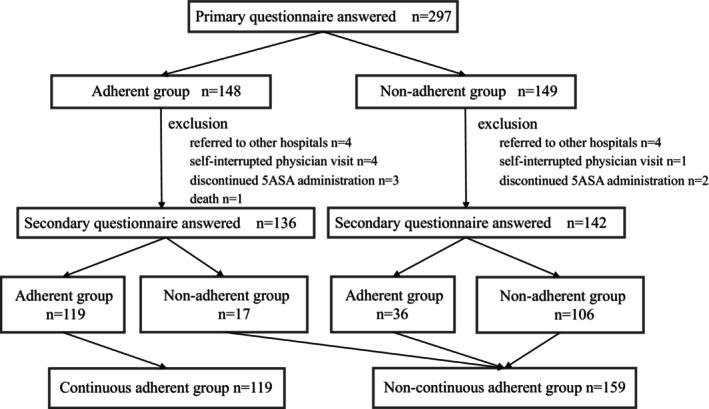
Results of the primary and secondary questionnaire survey over time. The continuous adherent group consisted of 119 patients (42.8%) who were adherent in both the primary and secondary questionnaire survey. In contrast, the non‐continuous adherent group consisted of 159 patients (57.2%).

**FIGURE 4:**
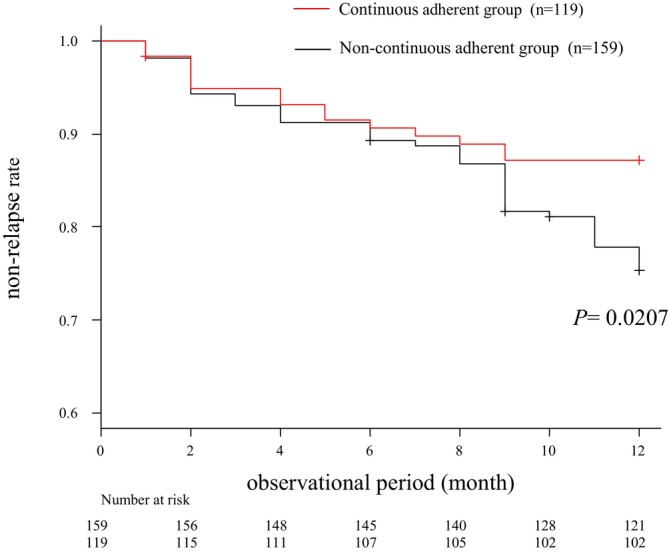
The cumulative non‐relapse rate after 1 year in the continuous adherent group (*n* = 119) and the non‐continuous adherent group (*n* = 159). The continuous adherent group was 85.7% and non‐continuous adherent group was 76.1% (*p* = 0.0207, Log‐rank test).

Figure 3 presents the results of the primary and secondary questionnaire surveys over time. The continuous adherence group comprised 119 patients (42.8%) who adhered to both the primary and secondary questionnaire surveys. In contrast, the non‐continuous adherence group consisted of 159 patients (57.2%). Clinical relapse occurred in 55 patients (19.8%), with additional induction therapy including 20 patients receiving a dose escalation of oral 5‐ASA, six patients starting a dose escalation of thiopurine, 11 patients starting prednisolone, 13 patients starting biologic agents, two patients starting calcineurin inhibitors, and three patients starting cytapheresis. Figure 4 shows the cumulative non‐relapse rates for both the continuous and non‐continuous adherence groups, and the non‐relapse rates 1 year after the initial questionnaire survey were 85.7% and 76.1%, respectively. Of the patients who were confirmed to be adherent at the first survey, 17 became non‐adherent at the second survey. Of the 17 patients, 5 had relapsed; the non‐relapse rate for these 17 patients was 70.6%. The cumulative non‐relapse rate was higher in the continuous adherence group than that in the non‐continuous adherence group (*p* = 0.0207).

## 4 Discussion

In the remission maintenance treatment of UC, 5‐ASA is recommended as the anchor drug [1, 2]. It is desirable to maintain remission for a long period in UC, which often shows a relapsing course, and 5‐ASA is used in most cases, except in patients who are intolerant.

The reasons for relapse in patients in remission maintenance with 5‐ASA agents include decreased responsiveness to 5‐ASA agents, insufficient dose, and non‐adherence, in addition to stress and infection as triggers. Of these, non‐adherence is a patient‐related factor that can be improved, except for intentional non‐adherence. Therefore, it is important to determine the frequency of non‐adherence and its factors in clinical practice. In previous reports, patients who took more than 80% of the prescribed dose were often defined as highly adherent, with adherence rates reported to be 40%–60% [3, 4, 5]. In contrast, in this study, patients who responded that they never missed a dose were strictly defined as adherent, and those who missed a dose once a week were strictly defined as non‐adherent. However, the adherence rates during the primary and secondary questionnaires were high, at 49.8% and 55.8%, respectively. This may be due to the fact that Japan has a universal health insurance system and a support system of medical cost for intractable disease with moderately to severe activity, resulting in low economic non‐adherence, and that our institution has an inflammatory bowel disease center where medical staff provides various patient education including adherence. Ethnicity has also been implicated in the differences in adherence rates to 5‐ASA [15].

Patient factors affecting adherence identified in this study included full‐time employment and a history of eating one or two meals per day. Although many studies have identified younger age, male sex, and full‐time employment as factors associated with lower adherence [13, 14, 15, 16, 17, 18, 19], this study found no significant differences in age or sex. In this study, full‐time employment was listed as an independent factor for low adherence. Ediger et al. and D'Incà et al. also reported an association between being employed and non‐adherence, possibly due to the increased time demands associated with employment [13, 14]. Also, the non‐carrying of medications to the workplace is conceivable. Regarding eating habits, there have been no reports of a relationship with UC, and Kato et al. reported that patients who sometimes skipped breakfast tended to be less adherent, but the difference was not significant [25]. Since medication is often indicated after meals, self‐interruption of medication due to not eating may be a possible cause. To improve adherence, it is important to pay attention not only to age and sex but also to occupational type and dietary habits.

The number of daily doses has the greatest impact on adherence to oral medications, not only 5‐ASA [9, 10]. A multicenter single‐blind study showed that once‐daily dosing was associated with significantly higher adherence and patient satisfaction in patients with UC taking 2 g of mesalazine once or twice daily. Furthermore, the remission maintenance rate was reported to be 70.9% for the once‐daily dose and 58.9% for the twice‐daily dose [11]. Likewise, the results of the adherence rate in the current study showed that the adherence rate for the once‐daily 5‐ASA was significantly higher than for the multiple daily doses. In this study, the percentage of patients receiving once‐daily 5‐ASA was significantly higher at the time of the secondary survey than at the time of the primary questionnaire, and in the 36 patients who switched from multiple dosing to single dosing at the time of the secondary survey, the adherence rate was significantly higher, from 41.7% to 72.2% (*p* = 0.0167). This suggests that the increase in once‐daily dosing may have contributed to the improvement in adherence. The number of daily doses of 5‐ASA varies for each drug; however, in recent years, once‐daily dosing has increased and should be recommended to patients. Furthermore, it has been reported that taking more than four medications per day and increasing the number of tablets taken at a time can decrease adherence [4, 12]. However, physicians should be aware that reducing the number of doses would simultaneously increase the number of tablets taken at one time. Regarding the formulation of 5‐ASA, Yagisawa et al. examined the acceptability of tablets and granules and reported that granules were more acceptable to patients [26]. The 5‐ASA granule formulations have a higher mesalazine purity and lower oral doses than the tablets. To improve adherence, patient preferences should be considered when selecting 5‐ASA formulations.

Although there have been previous reports on the association between adherence and disease activity, few prospective observational studies have examined adherence over time and the factors affecting disease relapse. This study examined activity over a one‐year period and prospectively observed clinical relapse in patients with well‐defined disease activity. In addition, the adherence status was checked twice at one‐year intervals to conduct a high‐quality analysis of the relationship between adherence and maintenance of remission. The results showed that the one‐year clinical non‐relapse rates were 85.7% and 76.1% in the continuous and non‐continuous adherent groups, respectively, with significantly fewer clinical relapses in the adherent group. Although the definitions of relapse and observation period differed, the results were similar to those of previous studies [3, 6]. Actually, this data does not accurately reflect adherence at relapse. However, of the patients who were confirmed to be adherent at the first survey, 17 patients became non‐adherent at the second survey. In spite of a short period of 1 year, 5 of these patients (29.4%) had relapsed. This fact suggested that it was important to maintain adherence in order to maintain clinical remission. In this study, adherence was strictly defined as not missing a dose, suggesting that more rigorous adherence to 5‐ASA is important for preventing clinical relapse.

This study has several limitations. First, this study was conducted at a university hospital, a high‐volume IBD center, and the patient backgrounds were different from those in general hospitals and clinics. Many patients have experienced severe flare‐ups in the past, and the results were not similar to those of the average patient with UC in Japan. Second, adherence was based on self‐reported data from a questionnaire survey, which could not be rigorously verified by checking for 5‐ASA metabolites, and it is possible that higher adherence was observed than what was actually the case. Although the patients answered in the waiting room where the physician was not present or at their home, the potential bias regarding explanation by physicians may have existed. Third, factors such as smoking status, educational level, and economic and health system‐related factors were not examined. Fourth, there were no specific medical staff interventions between the primary and secondary surveys. However, it is considered that the implementation of the first questionnaire survey may have served as a reminder to both doctors and patients about adherence to 5‐ASA. This study was able to prospectively examine factors related to adherence to 5‐ASA formulations and the relationship between adherence and clinical relapse in a relatively large number of patients; by conducting a follow‐up survey 1 year later, more detailed and reliable data could be obtained. The results of the follow‐up survey conducted 1 year later provided more detailed and reliable data and demonstrated that adherence was related to clinical relapse and the importance of adherence.

## 5 Conclusion

Adherence to 5‐ASA is associated with clinical relapse of UC, and strict adherence is important for maintaining remission. Factors associated with poor adherence to 5‐ASA preparations include the frequency of doses (multiple daily doses), eating habits (one or two meals per day), and full‐time employment. In the future, it will be important to reduce the number of doses administered and provide medication guidance and patient education, taking into account the patient's background, to improve adherence.

## Conflicts of Interest

F.H. received lecture fees from AbbVie GK, EA Pharma Co. Ltd., Janssen Pharmaceutical K.K., Mochida Pharmaceutical Co. Ltd., Mitsubishi Tanabe Pharma Co., and Takeda Pharmaceutical Co. Ltd., research grants from Eli Lilly Japan K.K., Janssen Pharmaceutical K.K., and scholarship grants from AbbVie GK, EA Pharma Co. Ltd., Otsuka Pharmaceutical Co. Ltd., Nippon Kayaku Co. Ltd., Mochida Pharmaceutical Co. Ltd., and Mitsubishi Tanabe Pharma Co. The other authors declare no conflicts of interest.

## Supporting information


**Figure S1:** Adherence rates for 36 patients who changed from multiple daily dosing during the primary questionnaire to once daily dosing during the secondary questionnaire. It was 41.7% (15/36) at the time of the primary questionnaire, but after changing to once‐daily dosing, it was significantly improved to 72.2% (26/36) at the time of the secondary questionnaire (*p* = 0.0167).

**Table S1:** Questionnaire survey form for patients with ulcerative colitis.
**Table S2:** Comparison of activity and blood test data in the Adherent group and the non‐adherent group at the time of the primary questionnaire.
**Table S3:** Comparison of patient backgrounds and 5‐ASA preparations in the Adherent group and non‐adherent group at the time of the secondary questionnaire.
**Table S4:** Comparison of activity and blood test data in the Adherent group and the non‐adherent group at the time of the secondary questionnaire.
**Table S5:** Multivariate analysis of factors related to adherence during the secondary questionnaire.

## Data Availability

The data that support the findings of this study are available from the corresponding author upon reasonable request.
