# Supplementary figures and images for: Prospective Questionnaire Survey on Adherence to Oral 5‐Aminosalicylic Acid in Patients With Ulcerative Colitis
Source: JGH Open. 2025 Aug 21;9(8):e70259. doi: 10.1002/jgh3.70259 (PMC12368352; doi:10.1002/jgh3.70259)

## Slide 1
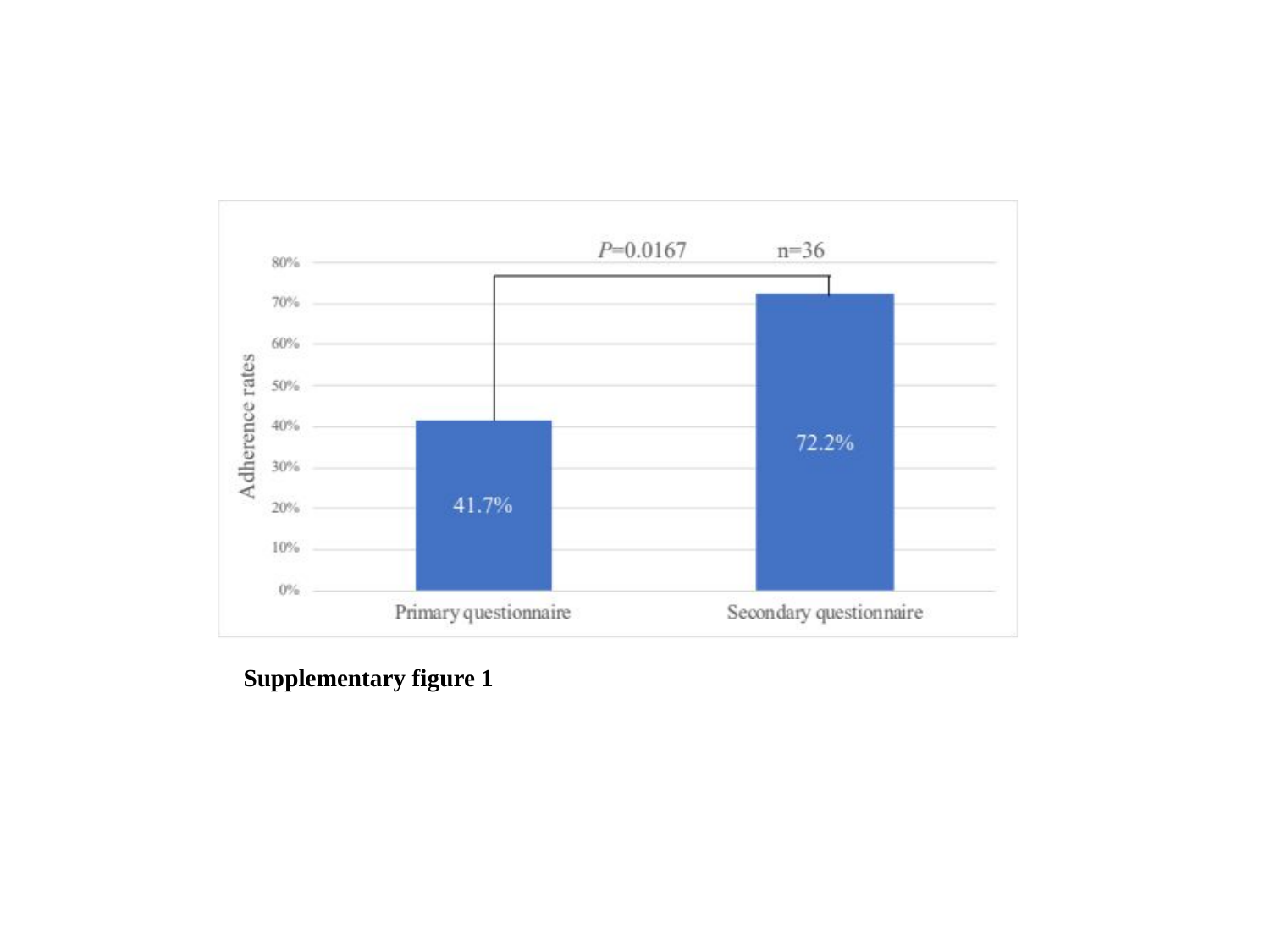

Supplementary figure 1

Supplement: Supplementary file 1 — Figure S1: Adherence rates for 36 patients who changed from multiple daily dosing during the primary questionnaire to once daily dosing during the secondary questionnaire. It was 41.7% (15/36) at the time of the primary questionnaire, but after changing to once‐daily dosing, it was significantly improved to 72.2% (26/36) at the time of the secondary questionnaire (p = 0.0167). [file JGH3-9-e70259-s002.pptx]
