# Supplementary material for: Prospective Questionnaire Survey on Adherence to Oral 5‐Aminosalicylic Acid in Patients With Ulcerative Colitis
Source: JGH Open. 2025 Aug 21;9(8):e70259. doi: 10.1002/jgh3.70259 (PMC12368352; doi:10.1002/jgh3.70259)
Supplement: Supplementary file 2 — Table S1: Questionnaire survey form for patients with ulcerative colitis. Table S2: Comparison of activity and blood test data in the Adherent group and the non‐adherent group at the time of the primary questionnaire. Table S3: Comparison of patient backgrounds and 5‐ASA preparations in the Adherent group and non‐adherent group at the time of the secondary questionnaire. Table S4: Comparison of activity and blood test data in the Adherent group and the non‐adherent group at the time of the secondary questionnaire. Table S5: Multivariate analysis of factors related to adherence during the secondary questionnaire. [file JGH3-9-e70259-s001.docx]

**Supplementary Table 1**

**Questionnaire survey form for patients with ulcerative colitis**

[Interview items]

Gender: □ Male □ Female

Age: □Under 15 years old □15-19 years old □20-29 years old □30-39 years old

□40-49 years old □ 50-59 years old □ 60 years old and over

Employment status: □ Student □ Full-time work □ Hourly work □ Housewife□ Others (including not

working)

Length of time since having this disease: □ Less than 1 year □ 1 year or more and less than 5 years

□ 5 years or more but less than 10 years □ 10 years or more

Current stage: □ active □ remission

Number of medicines taken: □ 1 type □ 2 types □ 3 types □ 4 types □ 5 types or more

Meal status: □ Always eats 3 meals □ Occasionally skips meals (→ □ Breakfast □Lunch □ Dinner)

Medical condition: Please fill in the average condition for the last 3 days

・Defecation frequency (number of times per day)

・Severity of bloody stools □ None □ Less than half of the time when defecation is slightly stained

with blood □ Obvious blood contamination during most stools □ mostly blood

・Abdominal pain □ None □ Yes (→ □ Mild □ Moderate □ Severe)

Oral 5-ASA formulation currently taken

Formulation name ,number of doses per dose,number of doses per day

Taking oral 5-ASA preparations “How much are you able to take?”

□ I take my medication regularly (morning, noon, and night) every day without fail.

□ Occasionally forget to take a dose, but less than once a week

□ Forgetting to drink once or twice a week

□ Forgetting to drink more than 3 times a week

→ If you were selected as forgetting to take, when do you often forget to take? (Multiple answers

allowed) □ Morning □ Noon □ Night (before going to bed)

**Supplementary Table 2**. Comparison of activity and blood test data in the Adherent group and the Non -adherent group at the time of the primary questionnaire

| Factors | Adherent group  (n=148) | Non -adherent group  (n=149) | *p* |
| --- | --- | --- | --- |
| Partial Disease Activity Index score  (mean±SD)  WBC ( / μl, mean±SD)  Hb (g /dl, mean±SD )  Plt ( × 10 ^4^ , mean ± SD)  Alb (g /dl, mean±SD)  CRP (mg /dl, mean±SD)  ESR (mm/h, mean±SD) | 0.65±1.11  5600.7±1936.8  13.7±2.0  25.2±7.4  4.4±1.4  0.22±0.6  18.8±19.1 | 0.81±1.19  5611.4±1809.4  14.1±1.6  24.7±6.4  4.4±0.3  0.17±0.5  15.8±15.4 | 0.223  0.961  0.088  0.543  0.776  0.447  0.134 |

*Note:* Values are presented as mean±SD.

Abbrevitations: SD, standard deviation; WBC, White blood cells; Hb, Hemoglobin.; Plt, Platelet;

Alb, Alubumin; CRP, C-reactive protein; ESR, Erythrocyte sedimentation rate.

**Supplementary table 3.** Comparison of patient backgrounds and 5-ASA preparations in the Adherent group and Non -adherent group at the time of the secondary questionnaire

| Factors | Adherent group  (n=155) | Non-adherent group  (n=123) | *p* |
| --- | --- | --- | --- |
| Gender (Male:Female)  Age  (Under 40:Over 40)  Disease duration  (Less than 5 years:5 years or more)  Disease type  (Pancolitis type:Others )  Working situation  (Full-time employment:Others)  Eating habits  (3 meals a day:1-2 meals a day)  Number of medications taken  (Less than 4 types:4 types or more)  5-ASA formulation  Dosage form (Tablet:Granule)  Number of tablets per dose  (Less than 3 tablets/packets:3 tablets/packets or more)  Number of doses per day  (Single dose:Multiple doses) | 93:62    47:108  35:120  77:78  76:79  125:30  112:43  124:31  75:80  56:99 | 70:53  45:78  23:100  63:60  77:46  81:42  100:23  99:24  60:63  26:97 | 0.625  0.305  0.460  0.810  0.0289  0.0059  0.0893  1.0  1.0  0.0080 |

*Note:* Values are presented as number.

Abbrevitation: 5- ASA, 5-aminosalicylic acid.

**Supplementary Table 4** . Comparison of activity and blood test data in the Adherent group and the Non -adherent group at the time of the secondary questionnaire

| Factors | Adherent group  (n=155) | Non -adherent group  (n=123) | *p* |
| --- | --- | --- | --- |
| Partial Disease Activity Index score  (mean±SD)  WBC ( / μl, mean±SD)  Hb (g /dl, mean±SD )  Plt ( × 10 ^4^ , mean ± SD)  Alb (g /dl, mean±SD)  CRP (mg /dl, mean±SD)  ESR (mm/h, mean±SD) | 0.53±0.76  5665.6±2146.5  13.6±0.1  27.2±19.0  4.3±0.4  0.39±1.6  17.8±15.8 | 0.56±0.87  5571.5±1728.3  14.0±1.6  25.9±7.4  4.4±0.3  0.17±0.3  14.5±15.1 | 0.757  0.694  0.184  0.468  0.103  0.145  0.077 |

*Note:* Values are presented as mean±SD.

Abbrevitations: SD, standard deviation; WBC, White blood cells; Hb, Hemoglobin.; Plt, Platelet;

Alb, Alubumin; CRP, C-reactive protein; ESR, Erythrocyte sedimentation rate.

**Supplementary table5.** Multivariate analysis of factors related to adherence during the secondary questionnaire

| Factors | Adjusted OR | 95%CI | *p* |
| --- | --- | --- | --- |
| Number of daily doses of 5-ASA preparations  multiple doses  Eating habits　　　 1- 2 meals a day  Working situation　　 full-time employment  Number of medications taken　 4 or more  Age 40+  Gender female  5-ASA dosage form granules | 1.95  2.09  1.93  0.76  0.81  1.46  1.14 | 1.02-3.75  1.18-3.70  1.13-3.30  0.38-1.52  0.47-1.38  0.85-2.50  0.56-2.31 | 0.044  0.0111  0.0155  0.431  0.427  0.166  0.454 |

Abbrevitations: 5- ASA, 5-aminosalicylic acid; OR, odds ratio; CI, confidence interval.
